# Correlation network analysis reveals relationships between diet-induced changes in human gut microbiota and metabolic health

**DOI:** 10.1038/nutd.2014.18

**Published:** 2014-06-30

**Authors:** T Kelder, J H M Stroeve, S Bijlsma, M Radonjic, G Roeselers

**Affiliations:** 1 Microbiology and Systems Biology, TNO, Zeist, The Netherlands

## Abstract

**Background:**

Recent evidence suggests that the gut microbiota plays an important role in human metabolism and energy homeostasis and is therefore a relevant factor in the assessment of metabolic health and flexibility. Understanding of these host–microbiome interactions aids the design of nutritional strategies that act via modulation of the microbiota. Nevertheless, relating gut microbiota composition to host health states remains challenging because of the sheer complexity of these ecosystems and the large degrees of interindividual variation in human microbiota composition.

**Methods:**

We assessed fecal microbiota composition and host response patterns of metabolic and inflammatory markers in 10 apparently healthy men subjected to a high-fat high-caloric diet (HFHC, 1300 kcal/day extra) for 4 weeks. DNA was isolated from stool and barcoded 16S rRNA gene amplicons were sequenced. Metabolic health parameters, including anthropomorphic and blood parameters, where determined at t=0 and t=4 weeks.

**Results:**

A correlation network approach revealed diet-induced changes in Bacteroides levels related to changes in carbohydrate oxidation rates, whereas the change in Firmicutes correlates with changes in fat oxidation. These results were confirmed by multivariate models. We identified correlations between microbial diversity indices and several inflammation-related host parameters that suggest a relation between diet-induced changes in gut microbiota diversity and inflammatory processes.

**Conclusions:**

This approach allowed us to identify significant correlations between abundances of microbial taxa and diet-induced shifts in several metabolic health parameters. Constructed correlation networks provide an overview of these relations, revealing groups of correlations that are of particular interest for explaining host health aspects through changes in the gut microbiota.

## Introduction

The microbial ecosystems in the mammalian gastrointestinal tract play an intricate role in animal health, including protective functions against pathogens, immune-system maturation and modulation of nutrient acquisition and host energy metabolism. Alterations in the composition of the gut bacterial communities have been implicated in metabolic disorders such as type 2 diabetes,^1^ metabolic syndrome,^2^ obesity^3, 4, 5^ and nonalcoholic steatohepatitis.^6^ Several studies suggest that specific bacterial phylotypes, or bacterial metabolic activities, could be beneficial or detrimental to patients with obesity.^5^ It is suggested that the proportional abundance of the most dominant bacterial phyla in the human gut, Bacteroidetes and Firmicutes, affect the efficiency of host energy harvesting and is linked with adiposity in both mice and humans,^5, 7^ although there is no consensus on these links.^8, 9, 10^ Nevertheless, it is clear that the gut microbiota delivers additional energy to the host in the form of short chain fatty acids (SCFAs); in the case of butyrate, it is reported to induce thermogenesis of the adipocytes.^11^ In turn, host diet and lifestyle have been shown to affect the gut microbiota.^12, 13, 14^ Together, these interactions with the host make the microbiota an intrinsic part of the system, maintaining the balance between (metabolic) health and disease. The gut microbiota is, therefore, a potential diagnostic, nutritional and pharmacological target in the management of obesity and obesity-related diseases.^4, 15, 16, 17^ To explore the relation between perturbations of the gut microbiota in diet-induced obesity and metabolic health factors, we studied the response to a high-fat high-caloric (HFHC) diet of a number of host parameters related to glucose metabolism, lipid metabolism, substrate oxidation (metabolic flexibility) and inflammation in relation to the composition of the gut microbiota. For this purpose, 10 male subjects were given a HFHCaloric diet for 4 weeks, and we monitored anthropomorphic, blood metabolic and health parameters, as well as the dynamic changes in the gut microbiota composition in stool samples before and after dietary intervention. The specific questions we aimed to address in this study were: (1) how does the distribution of microbial taxa change in response to HFHC diet-induced weight gain and (2) are specific members of the microbiota correlated with specific phenotypic characteristics, fasting levels of substrate oxidation and plasma markers?

To study the correlations in light of the host–microbiota system, we applied a network biology approach to identify clusters of correlating host parameters and microbiota. Network biology is an emerging field that represents biology as networks that capture the relations between the parts of a complex biological system, such as molecules, processes, organs or even different organisms.^18^ These networks provide a framework to aid understanding and modeling of the many interactions that maintain the balance between health and disease^19^ and are an invaluable tool to visualize and explore high-dimensional data sets.^20^ These aspects are especially relevant in studying host–microbiota interactions, given the high dimensionality of the resulting data sets and the complexity of the underlying host–microbiota system.

## Materials and methods

### Study subjects

Subjects were recruited from a pool of volunteers from TNO (Netherlands Organization for Applied Scientific Research, Zeist, The Netherlands). Ten apparently healthy men agreed to participate in the study. Inclusion criteria were: male sex, age 35–50 years, body mass index 23–30 kg m^−2^, having normal Dutch eating habits (consuming mostly three main meals including breakfast) and being a nonrestrained eater, defined as a score of <3.25 on the Dutch Restrained Eating Questionnaire.^21^ Exclusion criteria were: history of medical or surgical events that may affect the study outcome (including medication for diabetes, cholesterol-lowering medication, eating disorders and/or food allergies), smoking, consuming more than 28 units of alcohol per week and exercising for >3 h per week. Recruited subjects received a HFHC for 4 weeks. Response to the diet was measured at the beginning and end of this 4-week period.

### Ethics statement

Written informed consent was obtained from each participant after receiving an explanation of the procedures. Before the start of the study all subjects underwent a screening that involved an anamnesis about the medical history, lifestyle and eating behavior, measurement of body composition, blood pressure, waist circumference and collection of blood for clinical laboratory tests. The research protocol was approved by an independent medical ethics committee (The Medical Ethics Committee of Tilburg).

### Intervention study design

The HFHC diet consisted of a fixed amount of commercially available food items with high-fat and/or sugar content: candy bars, sausage rolls, coated peanuts and full-fat chocolate-flavored milk. Subjects received food items of the HFHC diet on weekly basis. Subjects were asked to maintain their habitual diet and lifestyle. On top of their habitual diet, subjects were asked to consume the items of the HFHC diet. The calculated nutritional composition of the weekly consumed HFHC items (28 food items) was 36 328 kJ, 215 g protein, 440 g fat and 965 g carbohydrates. This resulted in a daily surplus of 5190 kJ, 31 g protein, 63 g fat and 138 g carbohydrates. Subjects were instructed to divide their HFHC items equally over the course of the week. For the final week every subject received an extra snack-pack on top of the HFHC diet in order to maintain an increase in body weight. In order to monitor food intake and compliance, subjects were guided by a dietician on weekly basis. Before starting the diet, subjects were instructed to fill out a 3-day food record through which their baseline energy and macronutrient intake was calculated. In the first week of the intervention, compliance (based on returned food items) reached between 98 and 100%. In the second week, compliance was 100%, and in the third week, compliance was between 89 and 100%. In the final week, the compliance was between 95 and 100%.

### Physiological measurements

#### Indirect calorimetry and anthropometry

Substrate oxidation was measured using the *ventilated hood* method (QUARK RMR, version 9.1, Cosmed, Rome, Italy). The respiratory quotient (RQ) was assessed as the ratio of carbon dioxide exhaled divided by the amount of oxygen consumed by the individual (RQ=VCO2/VO2). At days 1 and 29, a measurement of 20 min was performed, reflecting the fasting substrate oxidation of the subjects. Data of the first 5 min were discarded. From the subsequent period of 15 min, a 10-min reading was selected that reflected a steady state.

Body composition was measured by a whole-body electrical resistance analyzer (InBody 720, Biospace, Seoul, Korea). Subjects' clothing was limited to underwear. Body weight, body fat mass, fat free mass, skeletal muscle mass and the visceral fat area were determined. Waist circumference was measured using a measuring tape 2 cm above the umbilicus.

#### Biochemical analyses

Blood samples were collected in ice-chilled tubes containing potassium ethylenediaminetetraacetic acid (K2EDTA) for plasma (Vacutainer Systems, Becton Dickinson, Plymouth, UK). Blood was centrifuged for 15 min at 2000 × *g* at 4 °C, within 15–30 min after collection. Plasma and serum were stored at −70 °C. Assays were performed at TNO using Olympus analytical equipment and reagents, except for adiponectin, insulin, cortisol, gastric inhibitory polypeptide (GIP), glucagon-like peptide-1 (GLP-1) and glucagon. GIP concentrations were determined by an enzyme-linked immunosorbent assay. Adiponectin, GLP-1 and glucagon were determined by radioimmunoassay. Insulin and cortisol were determined by an immunoenzymomatric assay. Plasma samples were used for multiarray analyses of seven inflammatory proteins, IFN-γ, IL-1β, IL-6, IL-8, IL-10, IL12p70 and TNF-α, using the Multiplex panel ‘Human Proinflammatory 7-plex' (Mesoscale Discovery, MSD, Gaithersburg, MD, USA) and of four vascular proteins, CRP, ICAM-1, VCAM-1 and SAA, using the Multiplex panel ‘Human Vascular Injury II' (Mesoscale Discovery).

SCFA and branched chain fatty acids were extracted from feces and quantified as described by Maathuis *et al.*^22^

### Feces sample collection and DNA extraction

Fresh stool sample were collected by subjects (*n*=10) at day 1 and day 29. Samples were frozen at −20 °C immediately by the subjects after defecation and immediately transported in frozen state to the laboratory at TNO where samples were mechanically homogenized, split into aliquots in sterile 2 ml cryovials and stored at −80 °C. Genomic DNA was isolated using the AGOWA mag Mini kit (DNA Isolation Kit, AGOWA, Berlin, Germany) according to the manufacturer's instructions.

### Pyrosequencing of barcoded 16S rRNA gene amplicons

A fragment of the 16S rRNA gene (∼330 bp), spanning the V5 and V6 hypervariable regions, was PCR amplified using primer 785F and 1061R as described previously.^23^ Purified PCR products were unidirectionally sequenced on a 454 Genome Sequencer FLX system (Roche, Branford, CT, USA) according to the manufacturer's protocols, resulting in 137 960 raw sequences. FASTA-formatted sequences and corresponding quality scores were extracted from the.sff data file generated by the GS-FLX-Titatium sequencer using the GS Amplicon software package (Roche). All data extraction, pre-processing, analysis of OTUs and classifications were performed using modules implemented in the Mothur software platform^24^ as in Roeselers *et al.*^25^ except where noted below. A total of 49 469 high-quality sequences were aligned using the ‘align.seqs' command and the Mothur-compatible Bacterial SILVA SEED database. A total of 4480 unique sequences were retrieved using this pipeline. OTUs were generated using a 97% sequence-identity threshold. Sequences were taxonomically classified by the RDP-II Naive Bayesian Classifier using a 60% confidence threshold. Community profiles were compared by Bray–Curtis dissimilarity and Weighted Unifrac clustering of OTU abundance.^26^ Sequences were normalized to 1000 sequences per samples (subsampling method). Population-level comparison of OTU abundance between stool samples collected before and after the HFHC diet was performed using the metastats tool.^27^

### Statistical analysis of host parameters

To test for difference in host parameters induced by the diet intervention, measurements at week 0 were compared measurements at week 4 using the analysis of variance model. For all analysis of variance measurements, the residuals plots were inspected. If these plots revealed a low variation for low values of the parameter of interest and a high variation for high values of the parameter of interest, the data were LOG transformed (=LN transformation). This transformation was performed on the original data set. If the residual was >3*√mse for a certain variable, the subject was considered as a statistical outlier and was removed from the particular data set. In all statistical tests performed, the null hypothesis (no effect) was rejected at the 0.05 level of probability. All analyses were performed in SAS 9.3 (SAS Institute, Cary, NC, USA).

### RV coefficient

The RV coefficient was calculated between the microbial and the physiological data. The RV coefficient is a multivariate generalization of the Pearson correlation coefficient.^28^

### Correlation network analysis

To quantify the change in microbiota parameters *M* (normalized microbiota abundance and associated ratios and diversity indices) in response to intervention (Δ*M*), the absolute difference before and after intervention were calculated as:


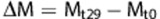


Where *M*_t0_ and *M*_t29_ are the values of a given microbiota parameter before and after intervention respectively. For the change in host parameters in response to intervention (Δ*P*) the relative difference between each parameter measurement before and after intervention was calculated as:


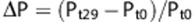


Where *P*_t0_ and *P*_t29_ is the measurement of a given host parameter before and after intervention respectively. Pairwise correlations between the Δ*M* and Δ*P* for each microbiota and host parameter were calculated using the Kendall tau rank correlation coefficient.^29^ Based on these correlation coefficients, a bi-partite correlation network was built where nodes represent either a microbiota or a host parameter. For each microbiota and a host parameter with an absolute correlation coefficient >0.6, an edge was added between the corresponding nodes in the correlation network. Correlations were calculated in R^30^ and the network was visualized in Cytoscape.^31^

### Multivariate statistical analysis

PLS^32^ was used to correlate the microbial composition/physiological data to several parameters including jackknife-based variable selection. For all models, the data were autoscaled to mean zero and unit variance. Leave-one-out cross-validation was applied; this may lead to optimistic results but it was the only possibility because of the limited number of available subjects in the study. Each PLS model resulted in a list of relevant parameters. All analyses were performed using Matlab R2012b (The Mathworks Inc., Natick, MA, USA) and the PLS toolbox for Matlab version 7.0.3 (Eigenvector Research Inc., Manson, WA, USA).

## Results

### Subject response to HFHC diet

**Figure 1:**
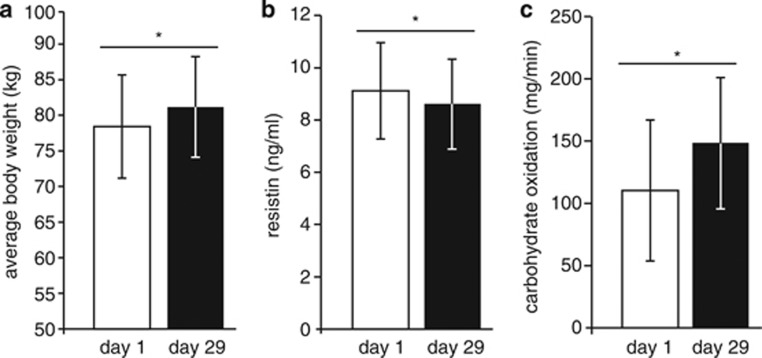
(**a**) Average weight gain, (**b**) blood resistin levels and (**c**) carbohydrate oxidation rates after 4-week HFHC diet intervention (*P*<0.0001, *n*=10).

The 4-week HFHC diet had a clear impact on the subject's body composition, with an average increase in body weight of 2.8 kg (*P*<0.0001; Figure 1), and resulted in increased fasting levels of markers of glucose and lipid metabolism. Supplementary Table S1 provides an overview of the observed diet-induced changes in the 96 measured host parameters, including phenotypic characteristics, fasting levels of substrate oxidation and plasma markers. Of these parameters, 26 were significantly changed (*P*<0.05) as a result of the diet intervention, including body weight, visceral fat mass, energy expenditure, and plasma insulin, cholesterol and leptin levels (Supplementary Table S2 and Supplementary Table S3C–E).

### High-throughput sequencing metrics

After all 137 960 raw DNA sequences obtained from the stool samples of subjects were subjected to rigorous quality control, a total of 49 469 high-quality, aligned sequences remained that were subsequently clustered into 980 unique operational taxonomic units (OTUs; 97%) and taxonomically classified into 115 genera within 8 bacterial phyla. The number of sequences per sample ranged from 1171 to 5420. Of these 49 469 pyrosequences, 16 805 were from stool samples collected before the HFHC diet (*t*=0) and 32 664 sequences were obtained from stool samples collected after the HFHC diet period (*t*=29). Although the sampling depth differed for these two groups, our sequence data set was large enough across both groups to permit comparison of the composition bacterial communities. Supplementary Tables S3A and B contain the relative taxonomic abundances for each subject.

### Relative abundances of microbial taxons

**Figure 2:**
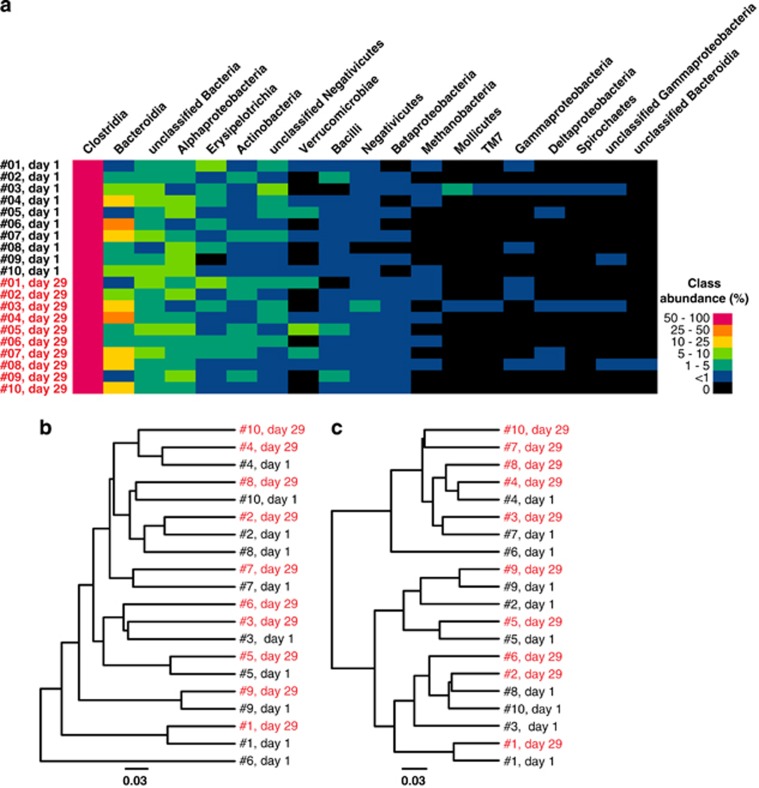
(**a**) Relative abundance of bacterial classes detected in each stool sample, represented as a heat map. (**b**) UPGMA dendrograms based on Bray–Curtis and (**c**) weighted Unifrac distances show that samples from a given individual type cluster mostly together. Samples collected at day 1 (*t*=0) did not cluster to the exclusion of samples collected at day 29 (*t*=29).

The microbiota comprised 8 bacterial phyla, of which 5 phyla were found across all samples, with the dominant phylum, based on relative abundance, in all samples being Firmicutes (77%) followed by Bacteroidetes (11%) and Proteobacteria (5%) (Figure 2a and Supplementary Figure S1). The proportions of Bacteroides with respect to Firmicutes varied among the subjects studied (Figure 2a). The Firmicutes–Bacteroidetes ratio was not statistically different between the samples collected before and after the HFHC diet.

### OTU-based microbiome population analysis

Next, samples were examined to determine how they grouped based on species diversity and OTU abundance. Samples collected at *t*=0 did not cluster to the exclusion of samples collected at *t*=29 (Figures 2b and c). Interindividual differences were apparent in the fecal microbiota and prevailed over potential diet-induced differences (Figures 2a–c). Population-level analyses showed that there were no OTUs that were significantly differentially abundant between stool samples collected before and after the HFHC diet.

### Overall correlation between the diet-induced changes in microbiota and host parameters

To assess the overall measure of correlation between the diet-induced changes in microbiota and host parameters, an RV coefficient was calculated. The RV coefficient of 0.368 was found between the relative abundance of microbial genera and host parameters, indicating a low overall correlation between the microbiota data and host parameters as a whole.

### Correlation network analysis of the diet-induced changes in microbiota and host parameters

Pairwise correlations between diet-induced changes in microbiota and host parameters (with an absolute Kendall coefficient of at least 0.65) resulted in a correlation network of 62 (out of 228) microbial parameters (taxonomic groups and two microbial diversity parameters) and 58 (out of 96) host parameters for which at least one correlation could be found. The network consists of 105 edges (correlations) and 120 nodes (microbial and host parameters) that clustered in 29 connected components (sub-networks). In total, 16 components consisted of 2 or more correlations, whereas the other components were single correlations between a host parameter and a microbial parameter (Supplementary Figure S2).

**Figure 3:**
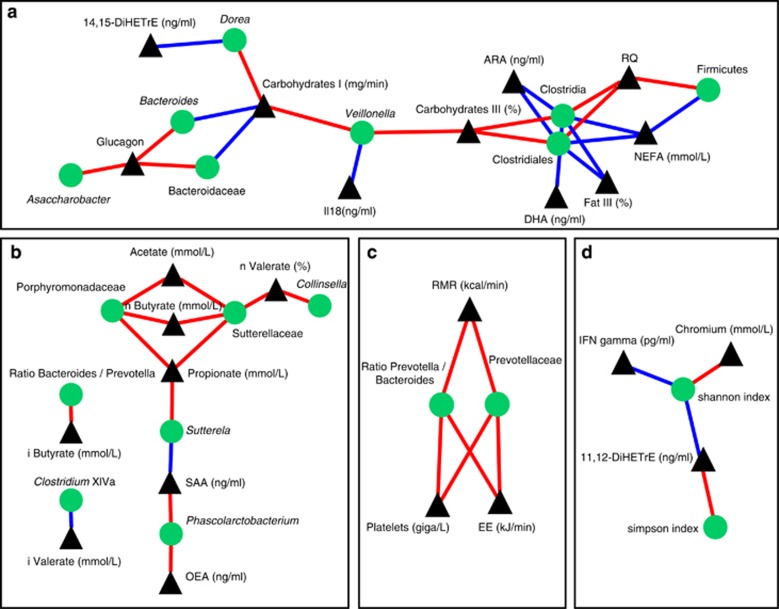
Four parts of the correlation network. (**a**) The largest connected component in the network. This component contains taxa within the phylum Firmicutes (Clostridia, Clostridiales, *Dorea*, *Veillonella* and Firmicutes itself), and taxa within the Bacteroidetes phylum (*Bacteroides*, Bacteroidaceae). (**b**) All correlations in the network involving SFCAs. (**c**) Connected component containing correlations with the *Prevotella*/*Bacteroides* ratio and host parameters. (**d**) Connected component of correlations of inflammation-related parameters with diversity indices. Black triangles represent host parameters and green circles represent microbial entities. Red and blue edges represent positive and negative correlations, respectively.

The largest component of the network mainly consists of taxonomic groups within the phylum Firmicutes (5 out of 8) and the phylum Bacteroidetes (2 out of 8) (Figure 3a). This component contains 10 different host parameters of which 9 are related to substrate oxidation (2 estimates for carbohydrate oxidation, 1 estimate for fat oxidation, the respiratory quotient, 1 pancreatic hormone regulating plasma glucose levels, 2 long-chain polyunsaturated fatty acids, 1 eicosanoid and total non-esterified fatty acid levels). The two estimates of carbohydrate oxidation (absolute measurement and % relative to fat oxidation) together correlate with most microbial taxa (six out of eight) that are exclusively from the Firmicutes and Bacteroidetes phyla. Notably, an inverse relation between the Bacteroidetes and Firmicutes was found, where all taxonomic groups within the phylum Firmicutes show a positive correlation with carbohydrates oxidation whereas all taxonomic groups within the phylum Bacteroidetes show a negative correlation. In addition, the Firmicute genus *Clostridium* and order Clostridiales were found to correlate positively with carbohydrate oxidation, but negatively with fat oxidation and the respiratory quotient (an index indicating the substrate use by the body ranging from 1.0, representing pure carbohydrate oxidation, to ∼0.7, representing pure fat oxidation), but negatively with fat oxidation, total non-esterified fatty acid levels and long-chain polyunsaturated fatty acids.

The second largest network component consists mainly of correlations with the SCFAs (four out of six parameters are SCFAs). For all SCFAs, a correlation with at least one microbial parameter was found (Figure 3b). All the identified correlations are positive (except for i-Valerate with *Clostridium* cluster XIV), implying a relative increase of these taxonomic groups with increasing SCFA concentrations. Both Porphyromonadaceae and Sutterellaceae correlate with multiple SCFAs (acetate, n-Butyrate, and propionate for both, and n-Valerate for Sutterellaceae only), whereas the other taxa (*Collinsella*, *Sutterela*, *Phascolarctobacterium* and *Clostridium* cluster XIVa) correlate to only a single specific SCFA. In addition, the ratio between *Bacteroides* and *Prevotella* abundance correlates with i-Butyrate.

The dietary intervention did not result in drastic changes in the *Prevotella*/*Bacteroides* ratio, as no significant change could be found in a groupwise comparison (*t*-test, *P*=0.843). Nevertheless, two host parameters related to energy balance, energy expenditure and resting metabolic rate, and blood platelet count were found to be positively correlated to the *Prevotell*a/*Bacteroides* ratio (Figure 3c). In addition, the abundance of Prevotellaceae, a family within the phylum Bacteroidetes, shows positive correlation to energy expenditure, resting metabolic rate and platelets.

For two of the three calculated microbial diversity indices, correlations with two inflammation-related host parameters were found (Figure 3d). The Shannon diversity index^33^ increases with increasing biodiversity, and negatively correlates with interferon-γ and 11,12-DiHETrE, an anti-inflammatory eicosanoid. The Simpson diversity index^34^ decreases with increasing diversity, and positively correlates with 11,12-DiHETrE, but not significantly with interferon-γ (*r*=0.31).

### Multivariate analysis of relations between changes in the microbiota and host parameters

To further investigate the identified groups of correlations between changes in microbiota composition and host parameters, partial least squares (PLS) models for selected correlations of interest were created. Five PLS models were created to investigate the relation between (1) *Bacteroides* and host parameters, (2) carbohydrates (mg min^−1^) and microbiota parameters, (3) Clostridiales and host parameters, (4) fat oxidation (%) and microbiota parameters, (5) energy expenditure and microbiota parameters, (6) *Prevotella*/*Bacteroidetes* ratio and host parameters and (7) the Shannon diversity index and host parameters. A valid fit could be obtained for 4 out of the 7 models, namely Clostridiales (*R*^2^=0.78), energy expenditure (*R*^2^=0.57), *Prevotella*/*Bacteroidetes* ratio (*R*^2^=0.89) and Shannon diversity index (*R*^2^=0.58). The regression values for the selected variables in each PLS model can be found in the Supplementary Data.

Next, parameters contributing to the multivariate PLS models were compared with the corresponding identified components in the correlation networks. For the Clostridiales model, all 5 out of 6 (all except fat oxidation) direct neighbors (direct correlations) in the correlation network were among the top 10 PLS model parameters. In addition, 8 additional parameters contributed to the PLS model, including 3 (9-HODE, 13-HODE and 12-HETE that can be released by lipoprotein lipase on the endothelium), an omega-3 fatty acid (EPA, synthesized from the essential fatty acid α-linolenic acid), body weight, adiponectin, leptin and the hematology parameters eosinophil counts and mean cell volume (a measure of the average red blood cell volume). The energy expenditure PLS model resulted in 85 selected microbiome parameters including its neighbors in the correlation network (Prevotellaceae and *Prevotella*/*Bacteroides* ratio), as well as changes in abundances of Prevotella and Bacteroides. The *Prevotella*/*Bacteroides* ratio PLS model resulted in 14 selected host parameters, including platelets, one of its neighbors in the correlation network. The PLS model on the Shannon diversity index contained 17 selected parameters that included all 3 direct neighbors in the correlation network. In addition, three parameters related to inflammation contributed to the PLS model (Complement C3, blood platelet counts and monocyte counts) as well as high-density lipoprotein cholesterol.

### Study limitations

The present study has limitations that should be acknowledged. Because of the absence of a control arm, we cannot exclude occurrence of spontaneous variations in microbiota composition and host parameters that were not related to the dietary intervention. The small sample size of this study (*n*=10) may have limited our ability to identify microbiota and host parameter features that could differentiate between subjects before and after the intervention. Further investigations are needed to evaluate our observed correlations between specific changes in microbiota composition and host parameters.

## Discussion

Overall, the physiological and biochemical response to a dietary perturbation is complex. By quantifying both a wide range of metabolic parameters and the gut microbiota composition before and after a 4-week HFHC dietary intervention, we generated a data set that allows us to make a step toward elucidating relations underlying this complexity. Using a correlation network approach, complemented by multivariate statistics, we charted the relations between changes in the microbiota and host parameters, and identified specific groups of correlations relevant to different aspects of metabolic health, such as substrate oxidation, energy expenditure and inflammation.

We identified a group of correlations enriched with diet-induced abundance changes of microbiota members. Specifically, we observed changes in the abundance of the phyla Firmicutes and Bacteroidetes correlated to changes in carbohydrate and fat oxidation rates in the host. This observation contributes to the ongoing debate on the balance between Firmicutes and Bacteroidetes in obesity.^5, 10^ Relations of this balance with body mass index have been identified in static conditions,^10^ and in response to long-term dietary restrictions.^5^ We report here a relation of changes in this balance with metabolic health in context of body weight gain by short-term (4 weeks) intervention. We did not observe a direct relation between Bacteroidetes and Firmicutes and body weight, likely because of the short time span of the intervention resulting in less profound body weight changes (2–5%) compared with the long-term intervention studies by Ley *et al.*,^5^ where correlations were observed on the range of 6–30% for the fat-restricted group. Nevertheless, we were able to detect relations with substrate oxidation rates that are indicative of diminished metabolic flexibility as a result of the intervention.^35^ This indicates potential for further research on monitoring changes in Bacteroidetes and Firmicutes balance as noninvasive marker for reduction in metabolic flexibility to identify risks for later stage disease phenotypes.

Arumugam *et al.*^36^ first articulated the concept of enterotypes as robust clustering of human gut community compositions, largely driven by the abundances of key bacterial genera.^36^ Although the distinction of enterotypes as either discrete clusters or a continuum will remains under debate, numerous studies have demonstrated the coexclusion of the closely related *Prevotella* and *Bacteroides* genera in the human gut microbiota.^37, 38^ Wu *et al.*^12^ suggest that this ratio is strongly associated with long-term diets, particularly diets rich in protein and animal fat (*Bacteroides*) versus carbohydrates (*Prevotella*). Our observed correlation between energy expenditure and the *Prevotella*/*Bacteroides* ratio supports this hypothesis and places this ratio in context of specific physiological factors in the host.

SCFAs, principally acetate, propionate and butyrate, are produced in the colon by bacterial fermentation of complex carbohydrates not digested in the small intestine.^39^ High fecal concentrations of total or individual SCFAs might be the result of increased microbial production, shifts in microbial cross-feeding interactions or reduced mucosal absorption. Nevertheless, it has been documented that changes in concentrations and proportions of specific SCFAs are correlated with changes in bacterial taxa.^10, 40^ In our study, all measured SFCAs were found to correlate with at least one microbial taxon, supporting a strong relation between diet-induced changes in microbiota composition. We observed a strong correlation between changes in the abundance of Porphyromonaceae and changes in fecal butyrate, propionate and acetate. Interestingly, some early studies from the early 1980s indicated butyrate production by *Porphyromonas* (former *Bacteroides*) strains.^41^ The observed correlation between Sutterellaceae and butyrate, propionate, valerate and acetate was less clear as SCFA production has not been documented for members of this family.

The identified correlations between two diversity indices and several inflammation related host parameters may indicate a relation between diet-induced changes in gut microbiota diversity and inflammatory processes that alter the gut habitat. A similar inverse correlation has been established in rats with 2,4,6-trinitrobenzenesulfonic acid-induced chronic colitis and in human subjects with Crohn's disease.^42, 43^ We observed that increasing diversity is associated with decreasing concentrations of the cytochrome *P*450 epoxygenase-derived eicosanoid 11–12-DiHETrE. In general, cytochrome *P*450 epoxygenase products have potent anti-inflammatory and vasodilatory effects that may indicate that subjects with a higher microbial diversity have a lower inflammatory response to the HCHF diet.

The HFHC intervention resulted in subtle and heterogeneous shifts in microbiota composition in which the interindividual variation in microbiota composition dominates over variation introduced by the interventions. In addition, the overall correlation in diet-induced changes between the total microbiota data set with the total set of host parameters was low. Nevertheless, by applying a correlation network approach we were able to identify relationships between specific changes in microbiota composition and host parameters such as substrate oxidation rates, energy expenditure and colonic SCFA concentrations. These observations highlight two strengths of the correlation network approach. First, rather than treating individuals as homogeneous group and looking for changes in mean parameter values, the correlations take into account the interindividual variations and focus on relations between parameters rather than requiring changes in group-wise means. In addition, rather than listing individual tables of correlations or correlating both data sets as a whole, visualizing all univariate correlations together in a network made it possible to identify and focus on smaller subsystems within the complete microbiota. Using this approach, groups of biologically related parameters emerge from the data, such as the immune/inflammation parameters correlating with microbial diversity. This provided a more integrated view on effects of dietary perturbation and resulting interaction between two complex systems.

This study provides insight into the host–microbiota system in the context of a short-term HFHC diet. Additional studies in different types of interventions and dynamic host data focusing on metabolic flexibility, such as time-resolved molecular measurements during challenge tests, will help to map and understand this system in a wider context of metabolic health. Network biology will provide key platforms for integrating and understanding these diverse types of data. On-going developments in this field focus on making networks more specific and powerful, for example by applying network deconvolution^44^ to limit the effect of spurious edges by indirect links. This will facilitate identification of microbial factors that may serve as early markers of changes in metabolic health that are predictive for long-term complications of metabolic syndrome. Network inference methods utilizing time-resolved information, such as methods for gene-regulatory network reconstruction,^45, 46^ may be applied to provide directionality to host–micriobiome interaction networks and discover causal links. This will facilitate discovery of intervention targets in the gut microbiota to improve human health.
