# Supplementary figures and images for: Correlation network analysis reveals relationships between diet-induced changes in human gut microbiota and metabolic health
Source: Nutr Diabetes. 2014 Jun 30;4(6):e122–. doi: 10.1038/nutd.2014.18 (PMC4079927; doi:10.1038/nutd.2014.18)

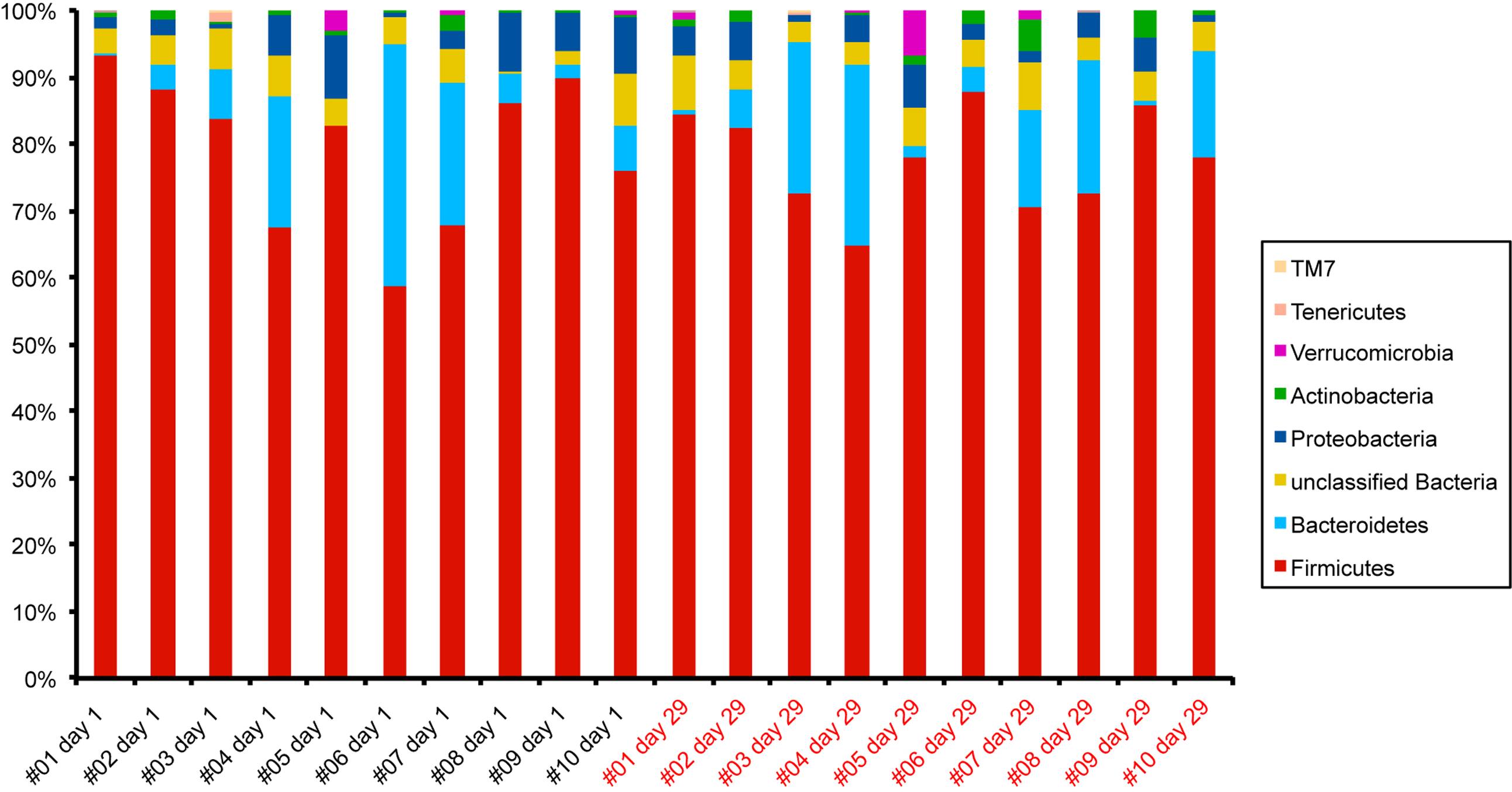

Supplement: Supplementary Figure 1 [file nutd201418x1.tif]

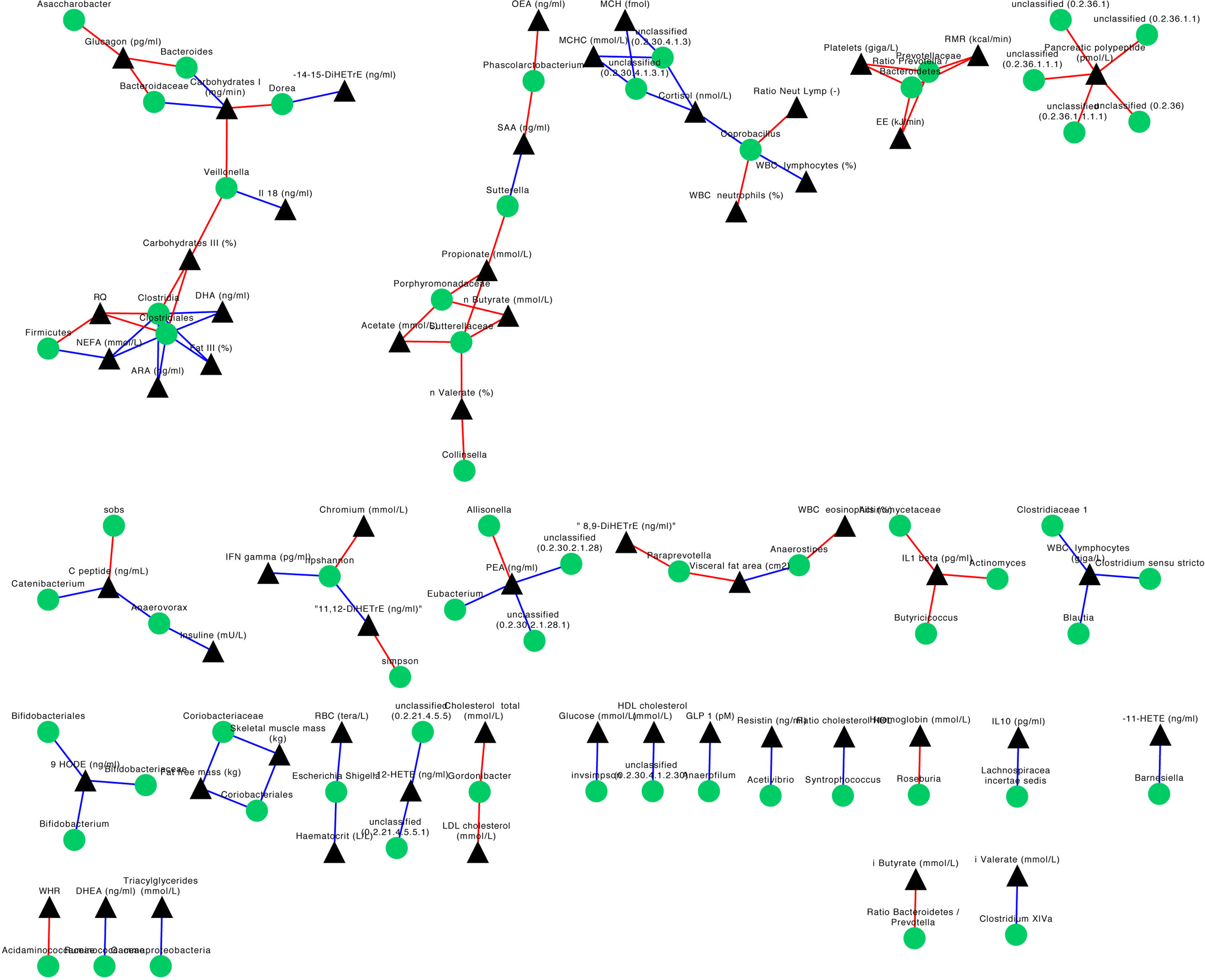

Supplement: Supplementary Figure 2 [file nutd201418x2.tif]
