# Supplementary material for: Correlation network analysis reveals relationships between diet-induced changes in human gut microbiota and metabolic health
Source: Nutr Diabetes. 2014 Jun 30;4(6):e122–. doi: 10.1038/nutd.2014.18 (PMC4079927; doi:10.1038/nutd.2014.18)
Supplement: Supplementary Figure and Table Legends [file nutd201418x6.doc]

**Correlation network analysis reveals relationships between diet-induced changes in human gut microbiota and metabolic health**

Thomas Kelder, Johanna H.M. Stroeve, Sabina Bijlsma, Marijana Radonjic, Guus Roeselers^*^

TNO, Zeist, The Netherlands

Corresponding author: [guus@roeselers.com](mailto:guus@roeselers.com)

## Supplemental Table & Figure legends

**Table S1.** Phenotypic characteristics and fasting levels of substrate oxidation and plasma markers before (day 1) and after (day 29) the 4-weeks high-fat high-calorie diet intervention.

**Table S2.** Significantly changed biomarkers categorized according to corresponding organs and process.

**Table S3.** Abundances of OTUs (97%) for each subject.

**Figure S1.** Relative abundance of bacterial phyla in each feces sample.

**Figure S2**: Networks of significant correlations between diet-induced changes in microbiota and host parameters.
